# Supplementary figures and images for: Histological characterization of denticulate palatal plates in an Early Permian dissorophoid
Source: PeerJ. 2017 Aug 22;5:e3727. doi: 10.7717/peerj.3727 (PMC5571816; doi:10.7717/peerj.3727)

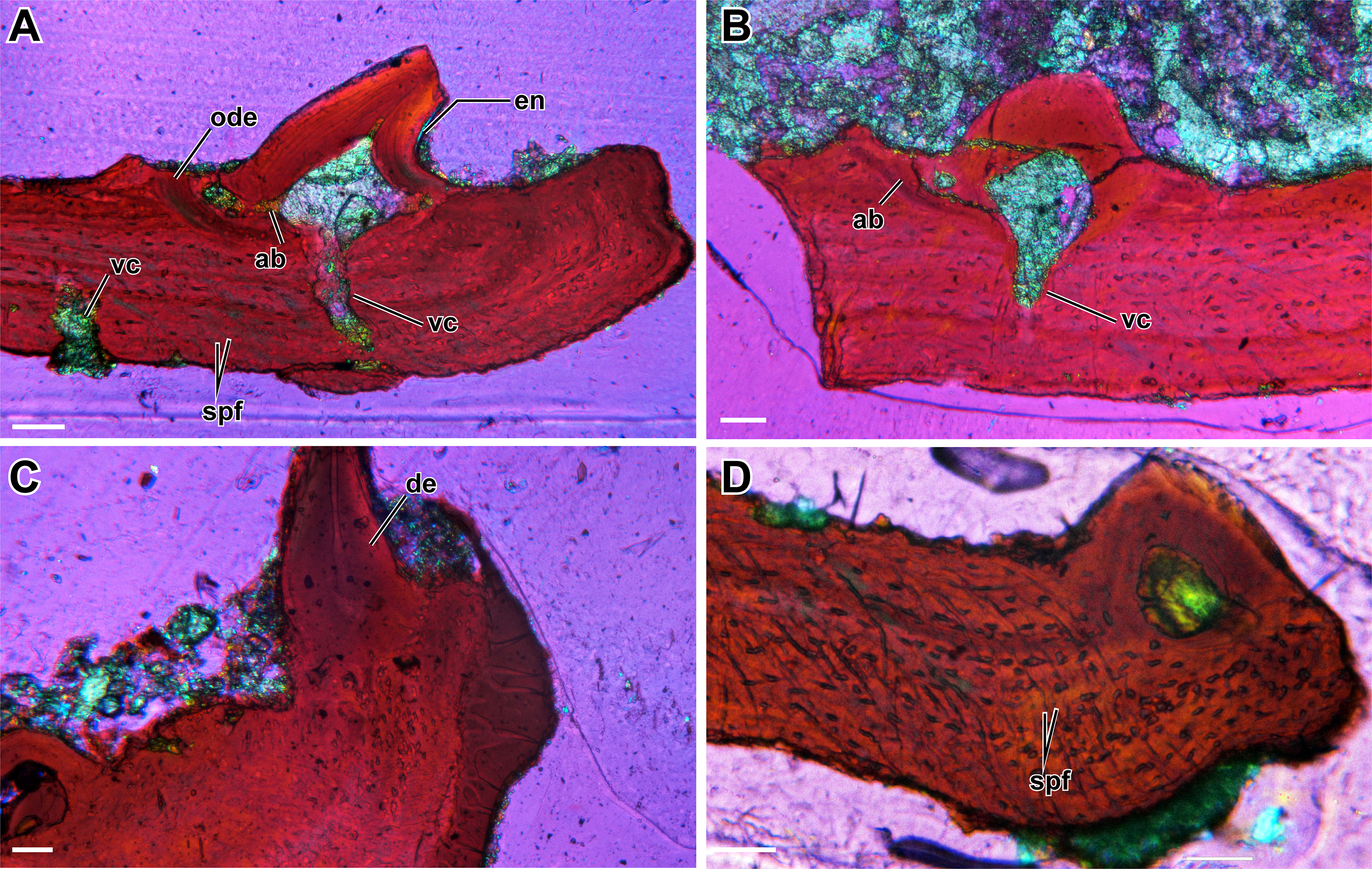

Supplement: Figure S1 — (A) TS01135; (B) TS01137; (C) TS01140; (D) TS01144. Scale bar = 500 µm. [file peerj-05-3727-s001.png]
